# Collegial Relationships

**DOI:** 10.1007/s10677-021-10165-9

**Published:** 2021-02-05

**Authors:** Monika Betzler, Jörg Löschke

**Affiliations:** 1 Faculty for Philosophy, Chair for Practical Philosophy and Ethics, LMU Munich, Geschwister-Scholl-Platz 1, D-80539 Munich, Germany; 2 Center for Ethics, University of Zurich, Zollikerstrasse 117, ZOA 211, CH-8008 Zurich, Switzerland

**Keywords:** Collegiality, Ethics of relationships, Work ethics, Relationship goods, Partiality, Solidarity, Recognition

## Abstract

Although collegial relationships are among the most prevalent types of interpersonal relationships in our lives, they have not been the subject of much philosophical study. In this paper, we take the first step in the process of developing an ethics of collegiality by establishing what qualifies two people as colleagues and then by determining what it is that gives value to collegial relationships. We argue that A and B are colleagues if both exhibit sameness regarding at least two of the following three features: (i) the same work content or domain of activity; (ii) the same institutional affiliation or common purpose; and/or (iii) the same status or level of responsibility. Moreover, we describe how the potential value of collegial relationships is grounded in the relationship goods that two colleagues have reason to generate *qua* colleagues, namely, collegial solidarity and collegial recognition. Two interesting conclusions that can be drawn from our analysis are that one has to be proficient at one’s work if one is to be considered a good colleague and that we are also more likely to be better colleagues if we regard the work we do as valuable. Finally, we draw special attention to the working conditions that are conducive to the generation of good collegial relationships and suggest some policies to promote them.

## Introduction

One of the most prevalent types of interpersonal relationships in our lives is the work relationships we have with our colleagues. As many of us spend more time with our colleagues than we do with our friends or family, colleagues can make a huge difference to the quality of our lives: we can look forward to going to work simply because we enjoy working with our colleagues or we can find it frustrating to have to put up with their shortcomings; they can contribute to and even inspire us in our work or they can make our workplace environment unpleasant, even unbearably so. As a result, workplace relationships between colleagues can have a significant impact on our lives.^1^ Psychological studies confirm that relationships with colleagues are an important contributor not only to job satisfaction but also to organisational performance.^2^ Collegiality is thus a significant factor in both individual and social well-being.^3^

Despite their significance, collegial relationships have not been the subject of much philosophical study. Whereas a great deal of attention has been devoted to other kinds of more intimate relationships, such as friendships, romantic partnerships and familial relationships,^4^ no systematic analysis of the relationship between colleagues has yet been undertaken. Some philosophers studying personal relationships have taken a brief look at relationships between colleagues, only to return to the more popular subject of intimate relationships.^5^

Given that workplace relationships between colleagues can have such a significant impact on our lives, they deserve to be studied in more detail. A philosophical account of collegiality involves both descriptive and normative elements: it needs to establish what qualifies two or more people as colleagues and it needs to provide an explanation for the potential value and normativity of relationships between colleagues. Once their distinct value and normativity are properly understood, we can also show more clearly why relationships between colleagues are such important contributors to individual and social well-being.

The value of collegial relationships, however, cannot be elucidated by applying insights from other kinds of relationships that we consider to be valuable because it differs in important regards. To highlight this difference, consider the special reasons that colleagues can justifiably be taken to have. These reasons differ from the special reasons generated in other types of relationships.^6^ Take the following three scenarios, for example:

Amy and Anna are friends. Anna’s boyfriend has just left her; she is crestfallen and believes that she will never be happy again. Amy spends many evenings empathising with Anna, trying to comfort her and bolster her self-worth as well as pointing out new life options to her.

Ben and Beth have been living together for 20 years. Beth has just been sacked. To make ends meet, they both need to work. The only new job Beth has been offered is in a town 800 km away from their home. Since Ben is unable to resign from his job and move with her, they decide that Beth will have to move away to take up her new post. To maintain their relationship, Ben and Beth take turns visiting each other at the weekends. They also plan to spend more quality time together, although this means that they will have to give up some of their hobbies and see their friends less often.

Clayton is the father of five-year-old Craig, who has just been diagnosed with autism. Craig has problems at school and needs additional support to help him deal with them. Clayton uses up most of his savings to find the best therapies for Craig’s condition. He cuts back his working hours to spend more time with Craig and he patiently puts up with his son’s occasionally challenging behaviour.

The descriptions of these scenarios suggest that Amy and Anna have a deep friendship, Ben and Beth have a strong romantic partnership and Clayton is a good father to Craig. One reason that these relationships are flourishing is that all the protagonists acknowledge that there are special reasons for acting well towards the other person in the relationship and so they each respond accordingly.

Clearly, the reasons colleagues have to behave in a certain way towards each other differ from the reasons of the participants in these other kinds of relationships. Colleagues are not expected to comfort each other when they break up from their partners (unless their work relationship has grown into a friendship); unlike romantic couples, they do not have to prioritise their relationship. A valuable collegial relationship also differs from a parent–child relationship in that colleagues are not expected to endure personal hardship to help a colleague who is not doing well. Nevertheless, our considered judgements suggest that colleagues do have special reasons to do things for each other that they would not have reason to do (or at least not an equally weighty reason to do) for a stranger. An ‘uncollegial’ colleague is someone who refuses to accept that these special reasons apply to her. The questions we then need to ask are how we can identify these special reasons and, relatedly, how they can shed light on the value of collegial relationships.

The aim of this paper is to identify the reasons colleagues have to give each other special treatment. This is a project in normative ethics, rather than, say, political philosophy: while questions such as workplace democracy are important and will be briefly discussed at the end of this paper, they are not our main concern here.^7^ Rather, by acquiring a better understanding of the special reasons that colleagues have for giving each other special treatment, we hope to contribute to the debate on reasonable partiality and the normativity of relationships. To do so, we first analyse what constitutes the particular *relation* that holds between colleagues in order to gain a better understanding of what makes two people qualify as colleagues. We then identify the distinct relationship goods that are generated by colleagues. Collegial *relationships* will flourish if colleagues accept that they have special reasons to realise these relationship goods. Two interesting conclusions that can be drawn from our account are that one has to be proficient at one’s job to be a good colleague and that we are more likely to be better colleagues if we regard the work we do as valuable. We conclude this paper by highlighting the practical implications of our view.

## What Is a Colleague?

The term ‘colleague’ is commonly used to describe people who work together. The Oxford English Dictionary thus defines ‘colleague’ as ‘a person with whom one works in a profession or business’.^8^ However, this lexical definition does not specify the exact nature of this work relation: the term ‘profession’ suggests that colleagues are involved in the same activities or share the same work content, while the term ‘business’ conveys that they share the same institutional affiliation. But it appears that not all the people with whom a person works in a profession or business should be regarded as colleagues. Superiors and subordinates, for example, also share some kind of work relation, although the parties in strongly asymmetrical power relationships are not considered to be colleagues.^9^ The term ‘colleague’ and the relationship it implies are also narrower than other terms that are commonly used to refer to people involved in a work relation, such as co-workers, employees, staff, team members or collaborators.

Throughout this paper, we will use the term ‘colleague’, rather than these other terms, for the following three reasons. Firstly, to be a colleague one has to be in a lateral peer relation: it takes some kind of shared status – however basic – for a person to qualify as a peer (in a shared activity or shared organisational context) and it requires some kind of equality or sameness to be in such a lateral peer relation. Even though the term ‘co-worker’ might be closest in meaning to the term ‘colleague’, the latter indicates more precisely some kind of equality or sameness that is characteristic of peers in a work environment. Secondly, unlike terms such as ‘staff’ or ‘employee’, the term ‘colleague’ implies that the parties involved relate to each other in second-personal ways that reflect an acknowledgement of their roughly equal status. Thirdly, and not unrelatedly, to refer to another person in one’s work environment as one’s colleague clearly suggests that one considers oneself to have special reasons to give that person special treatment. The usage of the term ‘colleague’ therefore does comprise the normative significance of the term that we are interested in. To strengthen this point, it is worth noting that, while the adjective of ‘colleague’ is ‘collegial’, there are no equivalent adjectives derived from the other kinds of abovementioned workplace relations.^10^ The relationship between two colleagues who consider themselves to be colleagues and take themselves to have special reasons to treat each other preferentially can be regarded as collegial. The term ‘co-worker’, by contrast, does not naturally carry such a normative meaning.^11^

But what is it exactly, that makes two persons qualify as colleagues? We have pointed out that the term ‘colleague’ reflects a lateral peer relation, but what conditions need to be fulfilled if this relation is to hold? We suggest that two people qualify as colleagues if they share: (i) the same work content or domain of activity; (ii) the same institutional affiliation or common purpose; and/or (iii) the same status or level of responsibility. Each of these features implies a certain kind of sameness.^12^

Concerning the work content or domain of activity (i), colleagues share particular tasks that can either contribute to the production of particular goods or to the execution of particular work-related activities at various levels of specificity. For example, carpenters and metalworkers can qualify as colleagues because they are both skilled manual workers and thus share manual labour, while locksmiths and welders qualify as colleagues because they are both metalworkers and work specifically with metal. A professor specialising in epistemology and a professor specialising in ethics both work in the field of philosophy, whereas a mathematics professor and a philosophy professor, even though they engage in different research and teaching subjects, share the broader pursuit of research, education and scholarship.

Sameness regarding institutional affiliation or common purpose (ii) typically pertains to membership in a particular organisational framework. This can mean working for the same employer or it can mean being a member of a broader institutional network, such as a professional organisation. For example, the secretary of ACME Corporation in France and the company’s Singapore-based accountant share the same employer. Two self-employed general practitioners might not have an employer, but they nonetheless share a broader institutional framework: they are both members of the same medical association that licenses them and determines the regulations which apply to them *qua* doctors. Furthermore, they work for the same common purpose, that is, to treat the sick and promote health, even in the absence of an institutional affiliation.

As for status and level of responsibility (iii), colleagues exhibit some sameness regarding their function within a particular organisational framework. That function is a result of the way in which they must ensure that others do their part to achieve the common goals of the particular organisation and how they contribute to achieving those goals themselves. For example, a carpenter and a locksmith might both be responsible for training apprentices. A professor of mathematics and a professor of philosophy have similar duties: they are responsible for carrying out certain professional services, including their own research work; and they also have the goal of ensuring that their students acquire and produce knowledge.

While all three features seem to capture something about the relation between colleagues, our considered judgements about particular cases do not suggest that people need to exhibit sameness with regard to all three features in order to qualify as colleagues. For example, people who share the same workplace but do not carry out exactly the same activities can still be regarded as colleagues. They exhibit sameness in relation to institutional affiliation (ii) and level of responsibility (iii) but not to work content (i).

However, two people do not seem to qualify as colleagues if they exhibit only one feature of sameness. Opinions may differ, but many would agree that a postdoctoral researcher in philosophy and the dean of a mathematics department are not colleagues, even if they belong to the same university. They neither share work content (i) nor level of responsibility (iii). Similarly, two chief executive officers (CEOs) from different branches of business who neither share content nor institutional affiliation can hardly be regarded as colleagues solely because they have the same level of responsibility. However, for the most part *any combination of two of these features typically qualifies two people as colleagues*. This also explains why the relation between three colleagues can be intransitive: if X is a colleague of Y, and Y is a colleague of Z, X is not necessarily a colleague of Z.

Some readers might reject our proposal, arguing that two people can qualify as colleagues if they work for the same company (or that two people can only be colleagues if they work for the same company), that is, they exhibit sameness exclusively in institutional affiliation. While we think that such an understanding of the term is vulnerable to counterexamples (Can the CEO of a company and the firm’s janitor really be regarded as colleagues?), we concede that the term is, to some extent at least, indeterminate.^13^ As far as we can see, none of the arguments we offer in this paper is affected by this indeterminacy and we can work with an understanding of the term that, for the most part, holds. Note, however, that our considerations so far only explain the *relation* that holds between colleagues.^14^ Two individuals can be in such a relation without knowing each other, without having any kind of contact with each other and without being aware of this relation. This explains why it is possible to meet someone who is your colleague even though you have never met that person before.

However, a relation that holds between colleagues is not the same thing as a relation*ship* that they might have with one another. The relation exists on account of the abovementioned features that colleagues have in common. It can, but need not, be the basis for a collegial relation*ship*. Two colleagues establish a relations*hip* based on the relation that holds between them by meeting and actualising interconnected and enduring behaviour chains in a work-related context.^15^ They can thus conduct this relationship in good or bad ways. Since a collegial relationship holds because of an antecedent collegial relation and since that relation exists because of the aforementioned features that two or more people share, it is plausible to assume that valuable collegial relationships also have something to do with the features that allow two people to qualify as colleagues. As we will argue in the following section on the value of collegial relationships, colleagues can generate particular relationship goods *qua* colleagues and these goods are conditioned by the features that they share with each other.^16^ As a result, there is a close connection between what it is that makes two people qualify as colleagues and what enables them to have a good collegial relationship.

## The Value of Collegial Relationships

The features that two colleagues have in common and which qualify them as colleagues help to explain and determine the ways in which colleagues can generate certain goods. These goods are relationship goods in the sense that only colleagues can realise them; and even if they were to be provided by people who are not colleagues, they gain special meaning and value when they are realised within the collegial relationship.^17^ Relationships between colleagues are valuable if the participants take themselves to have special reasons for generating these relationship goods.

Two collegial relationship goods stand out in particular: *collegial solidarity* and *collegial recognition*. These goods are multidimensional in the sense that they both have several aspects which demonstrate in what ways collegial relationships can flourish and hence be of particular value.^18^

*Collegial solidarity* is probably the more obvious good that colleagues have a special reason to provide within their relationship. An important dimension of solidarity is assistance^19^: when A shows solidarity with B, then A (attempts to) help B in a manner that is to be specified because of their perceived similarity.^20^ How exactly colleagues help each other depends on which dimensions of sameness they share and what makes them colleagues in the first place.

Take the case in which two people share the same work content and the same employer. It seems obvious that they can help each other out in ways in which only a colleague can. For example, Albert’s colleague Bertha might cover Albert’s shift for him so that he can take his sick child to hospital without having to worry about not fulfilling his work duties. This solidarity is a kind of assistance which people who do not share the same work content and the same employer cannot give. Certainly, most people would consider Berta to be a good colleague for taking over Albert’s shift in an emergency.^21^

Two people who do not work for the same employer but who can nevertheless be regarded as colleagues because they share the same work content and the same level of responsibility can also provide each other with a distinct kind of assistance. For example, Christine, a university lecturer, might read Doris’s paper and provide her with valuable feedback, thereby helping Doris to improve her arguments and subsequently increase the probability of her paper being accepted by a high-profile journal. This scenario is possible even if Doris and Christine do not work for the same department or university but have met at a conference and have subsequently sent each other drafts of their papers. Of course, Doris could get someone else to read her paper, but the feedback from a colleague has special value: it is much more likely to be relevant, because only a colleague can point out pertinent literature that she might have missed or flaws in her arguments as well as make helpful suggestions for improving the paper. Again, most people would regard Christine as being a good colleague for giving Doris this kind of feedback.

Colleagues can also show collegial solidarity with colleagues who do not share the same employer and who are even greater rivals than Christine and Doris. Suppose that Eric and Fred work for organisations that are direct competitors and that they meet at a trade fair where they have adjacent stands. Furthermore, suppose that Eric forgot to bring his notebook, which he needs for an important presentation, and that Fred volunteers to lend him his, since Fred knows what it is like to do business presentations and understands how awkward it is not to be able to give the expected power-point presentation. By lending Eric his notebook, Fred shows collegial solidarity and gives Eric collegial assistance: he helps him in a work-related matter because he is a colleague. Eric thus has a reason to regard Fred as a good colleague or to think that Fred acted in a collegial manner. Of course, cases of assistance among people who work for rival companies are often complex. If Carl’s solidarity with colleagues from another company jeopardises his own company – say, his help enables them to win a bid that his company would otherwise have obtained – then we would not call Carl a good colleague. By helping his colleagues who work for rival companies, Carl fails to show solidarity with those colleagues who are closer to him (we will return to this point below). But Carl’s reasons for showing collegial solidarity with a colleague from a rival company are outweighed by reasons for collegial solidarity of another kind, that is, the reasons for showing solidarity with the colleagues who work in the same company.

Hence, solidarity qualifies as an important collegial relationship good. It typically takes the form of a distinct kind of assistance against the backdrop of perceived similarities in the workplace. Collegial assistance can take many forms as its content can differ from case to case.^22^ This should come as no surprise: different colleagues can be colleagues as a result of the different features they share and, accordingly, they can assist each other in different ways. The value of this kind of assistance can increase, depending on the shared experiences and abilities of the colleagues. It is more valuable to receive assistance from a colleague than from another person in work-related matters, given that collegial assistance is built on these similarities.^23^

This demonstrates that we cannot truly understand what renders a relationship between colleagues valuable without first comprehending what qualifies two people as colleagues: the latter explains the kind of support that colleagues can give to each other. It is the determinants of their relation that enable them to produce particular goods in ongoing chains of interaction. What is more, collegial solidarity requires not only the willingness to help a colleague but also the ability to do so. We do not call somebody a good colleague if that person always appears ready to help out but lacks the ability to do so and thus constantly fails to be of any assistance.

However, this does not imply that collegial solidarity or its value is merely instrumental. Suppose that a group of academics in liberal countries organises a number of events to raise awareness of the situation faced by their colleagues in Turkey after the failed coup against the country’s president, Recep Erdoğan, in 2016. This action qualifies as collegial solidarity, even if the attempts to improve the working conditions of Turkish academics are unsuccessful. Collegial solidarity can be non-instrumentally good: those who unite in solidarity share the same endeavours and goals, and this can be valuable in itself. Or, to put things in somewhat Kantian terms, by attempting to provide collegial assistance, an agent makes the ends of another person in work-related matters her own ends, which is itself non-instrumentally good. It shows not only respect for the colleague but also respect for the work that they have in common: helping a colleague in work-related matters can send the message that the shared work is worth doing. Furthermore, solidarity among colleagues helps to alleviate the competitiveness that is often an inherent feature of work relationships. Collegial solidarity is thus not only instrumentally good but also non-instrumentally good.^24^

The second important collegial relationship good is *collegial recognition*: colleagues are in a unique position to assess and validate their work-related experiences and abilities. The difference between solidarity and recognition is not always clear-cut: some cases of collegial recognition might also count as collegial solidarity, but whereas solidarity is typically achieved while acting *on behalf of* one’s colleague, recognition is brought about by one’s behaviour *towards* one’s colleague.

Like collegial solidarity, collegial recognition concerns work-related matters and is based on the shared features that make two people colleagues in the first place.^25^ It also has several dimensions. Only colleagues can fully recognise each other’s professional skills, abilities and contributions to common work-related goals. Other people might also be able to appreciate someone’s professional skills – for example, you do not have to be a professional football player to recognise the athletic abilities of Lionel Messi – but it is usually more rewarding to have one’s work recognised by one’s colleagues. Only colleagues doing the same or similar type of work know from experience the specific demands and pressures of a particular job. This gives them the authority, which is based on their own work-related knowledge, to appreciate fully the work of their colleagues and thereby give them collegial recognition.^26^

A second important and connected aspect of collegial recognition concerns work-related experiences and can be explained by clarifying the distinction between ‘knowing that’ and ‘understanding why’. For example, other people can *know* by way of testimony *that* an agent will respond in a certain way to particular work-related matters, but it is only our colleagues who can truly *understand why* we will feel the way we do about a certain work-related experience. This recognition helps colleagues to acknowledge that their emotional reactions are justified.

Consider the following example. After several rounds of substantial revisions, Doris’s paper gets rejected by a high-profile journal. Doris is understandably disappointed. Christine, Doris’s colleague, and Eric, Doris’s spouse, are likely to respond very differently to this situation.

Eric will probably focus on the fact that Doris is crestfallen because he loves Doris and is concerned about her well-being. Since he knows her intimately and is familiar with her first-person standpoint, her interests, concerns and commitments, he can point out to Doris that her job is only one aspect of her many-faceted life. He will attempt to put things into perspective because he knows that Doris is extremely frustrated: he realises the importance of publishing papers because Doris shares her hopes and worries with him. However, Eric does not fully understand *why* she feels the way she does. He has never had an academic paper rejected after repeated rounds of revision. He thus has no clear understanding of the reasons for Doris’s disappointment.^27^

By contrast, Christine’s relationship with Doris revolves around their professional life. Consequently, even though their relationship is less intimate, Christine can understand more clearly why Doris is so disappointed. She will probably have experienced for herself the disappointment of having promising papers rejected and so – unlike Eric – need not infer what might cause such feelings. Hence, even if she cannot comfort Doris by pointing out the importance of other concerns and commitments that shape Doris’s life, she can ease Doris’s distress in other ways. She can empathise more easily with Doris as she can share and understand why Doris feels the way she does. She is thereby in a better position to recognise the appropriateness of Doris’s feelings. This enables her to offer a perspective that is close to their professional reality. Christine can tell Doris that the journal is known for its nepotism or that, despite the rejection of her paper, Doris is a good philosopher. Not only do these judgements pick out different considerations that might provide some comfort to Doris; they might even be better justified than Eric’s response precisely because they are rooted in shared professional practice, rather than in a loving partnership. Eric comforts Doris because he loves her, and he is concerned about what Doris cares about because he loves her. Christine comforts Doris because she has the same kind of work-related experiences as Doris. So, whereas Eric *knows that* Doris feels the way she does because of what she has said, Christine *understands why* Doris feels that way because she has had the same experience. This enables Christine to comprehend Doris’s disappointment more fully.

The kind of understanding that colleagues can offer each other is an important good. As agents with a perspective on the world, we have an interest in having people around us who can empathise with us, who can validate our first-person standpoint and who can understand what it feels like to be in our position. This relationship good is, of course, not limited to the academic professions. Colleagues who have the same boss share an understanding (both the negative and positive aspects) of what it is like to work for this person. They need not explain to each other why they feel anxious when they have to tell the boss that they have been unsuccessful in a certain task; they know that their colleagues will understand the uneasiness of the situation. Again, this is an important good for them because it involves recognising that their first-person standpoints are valid. By expressing their understanding of the stress caused by having to speak to the boss, a person’s colleagues acknowledge that this stress is justified.

Colleagues are, therefore, not only better able to recognise each other’s skills and abilities; they can also provide warrant to each other’s work-related experiences. And, like collegial solidarity, collegial recognition is directly linked to the particular nature of a collegial relationship. Two colleagues understand each other because of the shared features that make them colleagues in the first place: for example, colleagues understand why it is difficult to work for a specific company or in a specific field and know what it is like to have a specific level of responsibility.

The relationship goods account of collegiality has several interesting implications, two of which we would like to highlight. The first is that being a good colleague and being a loyal employee can be contradictory: solidarity with a colleague might require that one be disloyal to one’s shared employer (we will return to this below). The second and perhaps more surprising implication is that being a good colleague and being a friend can, in some cases, be incompatible. Collegial recognition might become less valuable when one’s colleague is also one’s friend, since a bias might come into play which leads to the recognition being based more on personal concerns than on professional standards. Since it seems unreasonable to claim that, as a general rule, colleagues should not become friends for professional reasons (although there might be circumstances in which colleagues might have a professional duty not to befriend each other), this incompatibility between being a good colleague and being a friend implies that being the best possible colleague is not something to which colleagues should necessarily aspire. There can be reasons for not being the best possible colleague.

Collegial solidarity and collegial recognition may not be the only relationship goods that colleagues can produce, but they are certainly two important ones that help to explain the value of collegial relationships. These goods can only be produced within the context of workplace relationships. And because collegial solidarity and collegial recognition are non-instrumentally good, people can justifiably feel a sense of loss when they lose their jobs and hence their collegial relationships. Furthermore, these relationship goods account for the contributive value that collegial relationships have for individual and social well-being.^28^ People are good colleagues when they help to produce these relationship goods; and since colleagues aim to have collegial and thus flourishing relationships with their colleagues, they have special reasons to help each other to generate these relationship goods.^29^ This explains why the relationship-dependent reasons that colleagues have differ from those that they have with friends and partners or parents and children. Generating collegial solidarity and collegial recognition does not involve comforting each other in matters of the heart or visiting colleagues at the weekends; it involves assisting and acknowledging each other in work-related matters. How strong these relationship-dependent reasons are in the case of colleagues depends on additional factors, such as how much one values a particular collegial relationship, what other morally relevant factors need to be taken into consideration and the conditions which hinder or foster the realisation of particular collegial relationship goods.

## Possible Objections and Implications

Finally, we would like to discuss some possible objections to the relationship goods account of collegiality. A first possible objection is that the work of people who want to be good colleagues will suffer as a result of their collegiality. Focusing on generating collegial solidarity and collegial recognition takes time and effort, so one could argue that it can lead to underperformance and poor-quality work.

Several things can be said in response to this concern. Firstly, an underperforming colleague might induce her colleagues to compensate for her shortcomings, a result that gives her colleagues more work not less. This explains, in part at least, why a colleague who does not do her work well is generally judged to be a bad colleague. Secondly, colleagues who do not even try to do a good job usually fail to respond adequately to the value of their work, which means that they fail to respond adequately to the value of their colleagues’ work. However, the goods of collegial solidarity and collegial recognition are based, in part at least, on the shared experiences relating to one’s work as well as on the shared experiences relating to the *value* of one’s work. Hence, colleagues who fail to respond adequately to the value of their work diminish the value of these relationship goods. As a result, in order to produce distinct collegial relationship goods, a colleague has to do well in her job; a collegial relationship with an underperforming colleague might, therefore, have little or no value.

However, these considerations could also lead to a second concern. If doing a good job at work is a precondition for producing collegial relationship goods, then this could exert normative pressure on people to overachieve. Suppose that John is passionate about his job and gives his work priority over the other aspects of his life. He is experienced in providing collegial solidarity and collegial recognition: he has intimate knowledge of his work and is highly skilled in assisting and acknowledging whatever is involved in the particular work he is doing. Does he thereby exert pressure on his colleague Jill to do the same? Does Jill have to keep pace with John if she wants to produce relationship goods with him? If the answer is ‘yes’, then collegiality would seem to be an inherently over-demanding concept in the matter of work performance.

We believe that one should not have to respond to the value of one’s work in such a demanding way in order to achieve collegiality. To be engaged in a successful collegial relationship one should respect one’s colleague as well as do one’s work well. This involves, in part at least, respecting the other colleague’s personal perspective and providing the relationships goods in ways that meet the particular colleague’s needs. Work is John’s top priority, but Jill’s perspective might involve several other engagements and projects. To produce relationship goods with Jill, John needs to provide his assistance in light of her particular situation and needs, for example by informing Jill about a meeting she missed because she had to pick up her children from school. Of course, given that Jill can only produce relationship goods with John if she does her work sufficiently well, she should not freeload. However, if John is to produce relationship goods with Jill, he must respect her personal perspective. This does not mean that John is prevented from excelling in his work; it simply means that John needs to acknowledge Jill’s particular situation, just as Jill should appreciate John’s excellence.

Up to this point we have assumed that the work which colleagues share is valuable. What happens, though, when the work undertaken has little or no value?^30^ If valuing one’s work is a precondition for collegiality, does that mean that ‘bad’ or unpleasant work makes collegiality impossible? Not necessarily. Just because there is no or very little reason to value one’s work, does not mean that one does not respect one’s colleague who is doing the same type of work. Indeed, colleagues who do the same type of ‘bad’ work can still produce collegial relationship goods, just as people who are engaged in the same struggle against an unjust cause can show solidarity with one another and give recognition to each other even though the context in which they do so has little or no value. The kinds of collegial relationship goods produced by colleagues undertaking work of little or no value includes sharing the burden of the bad work, showing mutual understanding for what it means to do that kind of work, supporting each other while enduring such hardship and striving for better working conditions. Collegiality might even become particularly important in such circumstances, since it introduces non-instrumental value to an otherwise valueless context.

But what if the work in which colleagues engage is not only bad but plainly immoral? Can mafiosi, for example, be regarded as good colleagues? Mafiosi should not help each other carry the burden of their work; they should simply stop what they are doing. There is clearly a difference between bad and immoral work. In the latter case, there are not only reasons not to value one’s work; there are also reasons to disvalue it. Bad work might still bring about some good and therefore have some product value, even though it has no performance value and does not provide the person undertaking it with a reason to value the work itself. Immoral work, by contrast, has negative product value. As a result, there are no reasons to continue doing immoral work, even if it has some performance value. People who undertake immoral work do not deserve any form of recognition and solidarity, only condemnation. Rather than regarding them as colleagues, mafiosi should be regarded as accomplices.^31^

If collegiality has non-instrumental value and can significantly contribute to people’s well-being, then clearly organisations should consider making working conditions more conducive to producing collegial relationship goods. This becomes even more prevalent in times of a pandemic, where such conditions are seriously thwarted and people are forced to work from home. For example, collegiality requires space, time and particular social structures. With regard to space, colleagues need room to meet, to converse, to have informal exchanges and to get to know each other. Cafeterias, restaurants, open spaces, gardens and meeting rooms as well as opportunities for career development programmes, social interaction and leisure activities are all ways of creating space. Colleagues also need to have the time to engage with each other. Regular shared breaks, an overlap in shifts for colleagues that leaves time for joint, after-work activities, as well as paid time for social interactions during work hours are all ways of fostering collegial relationship goods. As for social structures, unions are not the only institutions that can enhance collegial relationship goods by promoting the shared aims of people in the workforce. The ways in which an organisational culture relates to its collegial members are also important. Recognition of collegiality in performance assessments and promotion can help to reinforce collegial relationship goods. Furthermore, if managers value collegiality among their workforce, then they should also be collegial. This, in turn, should lead to a more egalitarian, supportive and ultimately more democratic workplace,^32^ which should also help to eliminate pernicious competitiveness and disrespectful domination.

To conclude, we would like to take a brief look at the genuine conflicts which can arise and to which we alluded earlier. For instance, although employees have special reasons to behave in a collegial manner, they also have role obligations towards their employer. What should be done in cases in which the role obligation that an employee has towards her employer clashes with the demands of collegiality?

One might think that, since employees have a role *obligation* towards their employer, whereas colleagues have (for the most part) merely special *reasons* to behave in certain ways towards one another, the former should always trump the latter in situations of conflict. But this is not necessarily the case. If the reasons for collegiality are to outweigh the employer’s interests, the latter must be justified on moral grounds. So, for example, if it is in the employer’s interest to thwart collegial relationships among the company’s employees because an atmosphere of intense competition and fear of being replaced leads to increases in profit, then this does not mean that the employees have a role obligation not to form collegial relationships. On the contrary, it means that the employer ought to stop prioritising profit over employee well-being.

In other cases, however, the employer’s interests can trump the reasons for collegiality (provided that the employer’s interests are morally justifiable). Take the abovementioned case of collegial behaviour towards colleagues that work for rival companies. We have already discussed cases in which the reasons for collegiality towards a colleague from another company conflict with the reasons for collegiality towards the colleagues of one’s own company. In this case, the latter reasons outweigh the former reasons, all else being equal. However, there might be cases in which an agent’s reasons for collegiality come into conflict with her role obligations towards her employer. In such cases, the interests of the employer seem to outweigh the reasons for collegiality: although helping a colleague from a rival company could be regarded as a good and praiseworthy thing to do, it usually does not rise to the level of a moral obligation. Instead, it seems supererogatory to help colleagues from rival companies in work-related matters (as long as they are not also one’s personal friends). On the plausible assumption that reasons for supererogatory behaviour cannot surpass one’s obligation, this means that, whenever helping a colleague comes into conflict with the justified interests of the employer, the latter should be considered to be more important.

But perhaps there are cases in which both the interests of the employer and the reasons for collegiality conflict with one another, without one of them having obvious priority over the other. A colleague might come into conflict with her employer, for example, without it being clear who is in the right, thereby leaving it up to the agent to decide who she should support: her colleague (and risk being branded a bad employee) or the employer (and risk being branded a bad colleague)? We must confess that we do not have a tried and true formula for resolving such conflicts; as is often the case in normative ethics, situations can arise where it is the agent, using her practical judgement, who must decide which of two competing claims are the more pressing. Morality is often messy and the demands of collegiality are no exception.

## Conclusion

The objective of this paper was to identify the special reasons to which collegial relationships give rise, and which help to make these relationships thrive and thus become valuable. We have argued that these reasons generate special relationship goods which colleagues can co-create by virtue of the shared features that make them colleagues in the first place. Two relationship goods stand out in particular, namely collegial solidarity and collegial recognition. If colleagues generate these goods, then their relationship is likely to flourish; and since colleagues have a vested interest in these goods, the latter should be instrumental in improving their lives. There is thus a close correlation between what makes two people colleagues, what it means for them to have a good collegial relationship and how this relationship can contribute to a good life.

The workplace relationship between colleagues is an important kind of social relationship that has been seriously under-theorised from a philosophical point of view. We hope, though, that in this paper we have made some progress towards developing an ethics of collegiality.^33^

## Footnotes

1 See Gaska and Frey 1996, 294, in which they point out that one of the main factors that contributes to a deterioration in a person’s well-being when that person loses her job or stops working is, other things being equal, the loss of relationships with colleagues.

2 See, e.g., Michaelson et al. 2014, 77–90. According to the Society for Human Resource Management’s (SHRM) 2016 Employee Job Satisfaction and Engagement Survey, workers named relationships with their co-workers as very important contributors to job satisfaction (40%) and as more important than the nature of the work itself. The most important factor (66%) mentioned is related to workplace relationships, namely the respectful treatment of all employees at all levels. See also Chiaburu and Harrison, 2008, 1082 –1103. See also Morgeson and Humphrey 2006; Van de Voorde et al., 2012, analysed 36 quantitative studies between 1995 and 2010 and were able to confirm the claim that employee well-being in terms of happiness and relationships is congruent with organisational performance.

3 See van de Voorde et al., 2012.

4 For recent accounts of the normativity of relationships in general, see Keller 2013, Seglow 2013 and Pettit 2015. However, none of these authors discusses collegial relationships in detail. Philosophical accounts of the normativity of friendship include Jeske 2001, Stroud 2006 and Keller 2007. Accounts of love and romantic partnerships include Kolodny 2003, Ebels-Duggan 2008, Helm 2010 and Jollimore 2011. Regarding the value of the family and the special obligations to which family relationships give rise, see Archard and Macleod 2002, Archard 2010 and Brighouse and Swift 2014.

5 See, e.g., Brighouse and Swift 2014, 104; Seglow 2013, 120 f.; Pettit 2015.

6 We will assume that the value of collegiality and the reasons colleagues have to be collegial are closely connected, but we do not subscribe to any particular theory concerning this connection. Some authors, such as Scheffler 1997, think that valuing a relationship involves the participants being willing to accept that the other party’s needs and interests generate special reasons for them. Others, such as Lazar 2016, maintain that relationships can be valuable and that it is an appropriate response to that value to regard the relationship as providing special reasons to act well towards the other party in the relationship. Either way, there seems to be a close conceptual connection between a valuable relationship and accepting that we have special reasons to act well towards the other party in the relationship.

7 For a philosophical account of workplace democracy, see Yeoman 2014 and Frega 2020.

8 See Oxford English Dictionary 2013. The term ‘colleague’ is used with the same root in all the Germanic, Romance and Slavic languages. The term comes from the Latin *collega* (from *com-* ‘together with’ plus *legare* ‘to choose’, which literally means one selected at the same time as another; or from *com-* ‘together’ plus *lex* ‘law’, which refers to two people being under the same law). See also Collins English Dictionary 2014. The term "college" was also used in the Roman Catholic Church referring to the papal governance of the church in collaboration with the local bishops whose  autonomy was respected. See Bunse 2001.

9 See Honohan 2001, 55.

10 The normative meaning of the term ‘colleague’ might explain why other authors who work on workplace relationships, but from a different perspective, do not use this term. For example, Estlund 2003 discusses workplace relationships largely from the perspective of legal and political theory, while Yeoman 2014 is mainly interested in workplace relationships from the perspective of political philosophy. None of these authors is interested specifically in the reasons that colleagues have to give each other special treatment.

11 This does not mean that vertical relationships, such as those between superiors and subordinates, are not valuable or normatively significant. We, however, are interested in the relationship between colleagues as lateral peer relationships. A detailed discussion of the value and normativity of vertical workplace relationships deserves to be examined in another paper.

12 We do not intend to provide a classical definition of the term ‘colleague’. As with other social role concepts, such as ‘friend’ or ‘family member’, the boundaries surrounding the application of the term are blurry. For our purposes, we start with a concept that provides us with a plausible explanation as to what colleagues in paradigmatic cases share.

13 For empirical studies that support our proposal, see Reuter, Löschke and Betzler 2020.

14 Compare Kolodny 2003, 147, and Seglow 2013, 27, on the concept of ‘relation’. See also Neuberger 1996, 269.

15 See Hinde 1996, 9: ‘A *relationship* involves a series of […] interactions between individuals who know each other, such that each interaction is affected by preceding ones and usually by the expectation of future interactions […].’ See also Seglow 2013*,* 28 f.

16 We have drawn here from Keller 2007, Seglow 2013, Brighouse and Swift 2014 and partly from Pettit 2015.

17 We focus on these relationship goods as we are interested in what makes collegial relationships intrinsically valuable for their participants. Of course, this is not to deny that collegial relationships cannot also be instrumentally valuable, both for their participants and for society at large. For an account of how collegial relationships can strengthen civil society, see Estlund 2003.

18 We thank Jonathan Seglow for clarifying the distinct relationship goods under discussion in collegial relationships.

19 We use a broad notion of solidarity which is not limited to ‘working with others for common political aims’ (Kolers 2012, 367) but encompasses ‘enacted commitments to accept costs to assist others with whom a person or persons recognise similarity in a relevant respect’ (Prainsack and Buyx 2018, 588).

20 See also Löschke 2015.

21 In what follows we test our considered judgements about particular cases in light of both the relationship goods that we discussed and the background theories pertinent to work relationships, and then bring them into reflective equilibrium. We thereby draw from the method of ‘wide reflective equilibrium’. See, e.g., Daniels 1996, 21–46.

22 Kram and Isabella 1985, 116 ff., have identified three types of colleagues, namely the ‘information peer’ and the ‘collegial peer’, who both engage in joint career strategising and job-related feedback, and the ‘special peer’, who provides emotional support, personal feedback and friendship.

23 See Sunstein and Ullmann-Margalit 2001, 135, and their account of so-called ‘fraternity goods’, which makes plausible the idea that the value of collegial assistance rises when it is provided by people belonging to a particular group. Colleagues can also acknowledge their respective social identities – such as class, race or gender – and the bearing these have on their work environment and assist each other in these respects in the workplace.

24 Collegial solidarity can also take the form of a sense of belonging, which is also non-instrumentally valuable for human beings as social creatures. For a discussion of collegial solidarity which also highlights this dimension, see Estlund 2003.

25 Ikäheimo 2002, 456, has identified three ‘objects’ of recognition: we can recognise (1) singular human beings with regard to their happiness and well-being; (2) a person’s autonomy and thus her rights; and (3) a person and her particularities as being worthy of esteem. All three senses of recognition can be involved in collegial recognition.

26 See Ikäheimo and Laitinen 2007, and Brandom 2007, 136, for similar considerations with regard to recognition.

27 For a detailed discussion of understanding *why* as a distinct epistemic state, see Hills 2016.

28 See footnotes 2 and 3.

29 Generating these goods involves having the ability to recognise, in particular cases, what is *not* involved in producing these goods. Our thanks to Luke Russell for pointing this out.

30 Our understanding of valuable work relies heavily on Clark 2017, 64ff., who argues that a distinct kind of pleasure, developing skills and democracy are the three factors that determine what qualifies as good work.

31 Soldiers are another interesting and more intricate example. Typically, we regard them as comrades rather than as colleagues or accomplices, which is possibly related to the fact that soldiers undertake morally tainted but not totally immoral work.

32 Frega 2020 argues that a democratic workplace is characterised by relational parity, inclusive authority and social involvement. Collegial relationships are particularly conducive to these goals.

33 We are particularly grateful to Marko Konjovic, Jonathan Seglow, Shanna Slank and Martin Sticker for their insightful written comments on earlier versions of this paper and we would also like to thank Luke Russell and Simon Keller whose inspiring discussions helped us enormously. We presented this paper to a number of audiences – at the conferences on ‘The Moral Demands of Relationships’ and ‘Neglected Relationships’ (both of which took place at the Ludwig Maximilian University of Munich, Germany), at a workshop given by Monika Betzler at the University of Belgrade, Serbia (at which Marko Konjovic provided valuable comments) and at the ‘Goldegger Dialoge’ at Goldegg, Austria – and appreciate the helpful feedback we received. We are also indebted to Margareta Simons for her excellent editorial help. Jörg Löschke’s contribution to this paper was supported by the Swiss National Science Foundation as part of the research project on ‘Value-Based Non-Consequentialism’ (Grant Number PP00P1_176703).
